# Draft Genome Sequence of an Actinobacterium, *Brachybacterium muris* Strain UCD-AY4

**DOI:** 10.1128/genomeA.00086-13

**Published:** 2013-03-21

**Authors:** Jonathon R. Lo, Jenna M. Lang, Aaron E. Darling, Jonathan A. Eisen, David A. Coil

**Affiliations:** University of California at Davis, Genome Center, Davis, California, USA

## Abstract

Here we present the draft genome of an actinobacterium, *Brachybacterium muris* UCD-AY4. The assembly contains 3,257,338 bp and has a GC content of 70%. This strain was isolated from a residential bath towel and has a 16S rRNA gene 99.7% identical to that of the original *B. muris* strain, C3H-21.

## GENOME ANNOUNCEMENT

Members of the *Brachybacterium* genus have been found in a variety of environments, including salt-fermented seafood (1), poultry deep litter (2), and deteriorated medieval wall paintings (3). *Brachybacterium* bacteria are characterized as Gram positive, nonsporulating, and nonmotile. They are coccoid shaped during the stationary phase and oval rod shaped when transferred to fresh medium (2).

*Brachybacterium muris* strain UCD-AY4 was isolated from a residential bath towel in Davis, California, in an effort to provide microbial reference genomes from the built environment. Fibers from a residential bath towel were incubated overnight in Luria broth (LB) at 37°C and then plated out on LB agar. Single colonies were picked for serial dilution streaking and the organism was identified by Sanger sequencing of the 16S PCR product produced by the 1391R and 27F primers. Genomic DNA was extracted using a Wizard genomic DNA purification kit (Promega) from fresh overnight cultures. Illumina paired-end libraries were then made from sonicated DNA using a TruSeq DNA sample prep v2 kit (Illumina). Fragments between 300 and 600 bp were selected using a Pippin Prep DNA size selection system (Sage Science). A total of 2,016,086 paired-end reads were generated on an Illumina MiSeq, at a read length of 160 bp. Quality trimming and error correction of the reads resulted in 1,997,827 high-quality reads. All sequence processing and assembly was performed using the a5 assembly pipeline (4). This pipeline automates the processes of data cleaning, error correction, contig assembly, scaffolding, and quality control. The assembly initially produced 107 contigs, contained in 42 scaffolds (minimum, 453 bp; maximum, 411,879 bp; *N*_50_, 221,825). During scaffolding some contigs were merged based on short overlaps and read pair information, yielding a final collection of 45 contigs in 42 scaffolds that were submitted to GenBank. This resulted in a final assembly of 3,257,338 bp with a GC content of 70% and an overall coverage estimate of 98×. Completeness of the genome was assessed using the Phylosift software (A. Darling, G. Jospin, E. Lowe, E. Matsen, H. Bik, and J. Eisen, unpublished data), which searches for a list of 40 highly conserved, single-copy marker genes (D. Wu, G. Jospin, and J. Eisen, unpublished data), of which all 40 were found in this assembly.

Automated annotation was performed using the RAST annotation server (5). *B. muris* strain UCD-AY4 contains 2,883 predicted protein-coding sequences and 53 predicted noncoding RNAs. A full-length (1,512 bp) 16S rRNA sequence was obtained from this annotation and was used to identify the species of *Brachybacterium*. A phylogenetic tree was produced using the Ribosomal Database Project (RDP), which implements a weighted neighbor-joining tree-building algorithm (6). In this tree, *B. muris* UCD-AY4 falls within a monophyletic clade of *B. muris* strains with 100% bootstrap support (doi: 10.6084/m9.figshare.628054). This sequence was 99.7% identical to *B. muris* C3H-21 isolated from mouse liver (7).

The genome sequences of only two other *Brachybacterium* species have been published, *B. faecium* (8) and *B. squillarum* (9). The 16S rRNA gene from *B. muris* UCD-AY4 has 97% identity to that from *B. faecium* and 98% identity to that from *B. squillarum*.

### Nucleotide sequence accession numbers. 

This whole-genome shotgun project has been deposited at DDBJ/EMBL/GenBank under the accession number AORC00000000. The version described in this paper is the first version, number AORC01000000. Illumina reads available at http://dx.doi.org/10.6084/m9.figshare.157178.
